# Development and Validation of a Three-gene Prognostic Signature for Patients with Hepatocellular Carcinoma

**DOI:** 10.1038/s41598-017-04811-5

**Published:** 2017-07-17

**Authors:** Binghua Li, Wendu Feng, Ouyang Luo, Tiancheng Xu, Yajuan Cao, Hongyan Wu, Decai Yu, Yitao Ding

**Affiliations:** 1 Department of Hepatobiliary Surgery, the Affliated Drum Tower Hospital, Medical School of Nanjing University, Nanjing, 210093 China; 2 Department of Pathology, the Affliated Drum Tower Hospital, Medical School of Nanjing University, Nanjing, 210093 China

## Abstract

Hepatocellular carcinoma (HCC) is the leading cause of cancer-related death worldwide, because recurrence often occurs in most HCC patients undergoing hepatectomy. It is necessary to identify patients with high risk for recurrence and adopt effective therapies. An obstacle to monitor patients at high risk for poor prognosis has been the lack of useful predictive biomarkers. Fortunately, recent progress in system biology allows to screen the biomarkers for HCC prognosis in a high-throughput manner. In this study, we performed systematic Kaplan-Meier survival analysis of the whole mRNA transcriptomics based on the Cancer Genome Atlas project (TCGA) and developed a three-gene prognostic signature composing of three genes UPB1, SOCS2 and RTN3. The model was validated in two independent microarray data sets retrieved from Gene Expression Omnibus (GEO) and the expression pattern of these three predictive genes in HCC was confirmed by western blot and immunohistochemistry with our HCC samples. In conclusion, our results showed that this three-gene signature has prognostic value for HCC patients.

## Introduction

Hepatocellular carcinoma (HCC) is estimated to be responsible for about 700,000 deaths globally in 2012, making it the leading cause of cancer-related death worldwide^1, 2^. With the development of non-invasive radiological techniques and the novel advancements in the management of HCC, at-risk populations such as hepatitis virus infected patients are often under close surveillance^3–5^. As a result, HCC is increasingly diagnosed at earlier stages and treatment is likely to be successful. Nevertheless, recurrences eventually occur in most patients undergoing hepatectomy and the outcome of HCC patients is still unsatisfactory^6, 7^. It is necessary to monitor patients at high risk for bad clinical outcome and adopt effective strategies to prevent recurrence. Therefore, identification of novel prognostic biomarkers is as important as early diagnostic molecular markers.

Traditional serum markers particularly alpha-fetoprotein (AFP)^8^ and AFP mRNA^9^ have been found to be prognostic. However, they rely on significant tumor burden making their usefulness in operable tumors questionable^10^. The past decades have seen the rapid development of predictive biomarkers with advances in understanding of tumor biology, and there is a large volume of published studies describing the role of tissue and serum markers such as tumor-associated antigens, oncogenes and tumor suppressor genes, enzymes and isoenzymes, adhesion molecules, angiogenic factors, growth factors and their receptors, tumor microenvironment, the microRNAs (miRNAs) and long noncoding RNAs (lncRNAs) in HCC prognosis^11–16^. Our previous study has demonstrated that the expression of glutaminase is a sensitive and specific biomarker for the prognosis of HCC^17^. However, traditional screening methods focused on few genes, suffered from a lack of systematical evaluation and single biomarker for prognosis may lack sensitivity and specificity. Fortunately, recent advances in high-throughput technologies allow quantitative monitoring of various biological molecules and make it much easier to examine a large number of potential biomarkers at once, leading to an explosion of new biomarkers for HCC prediction. The innovative and seminal work of Hoshida discovered and validated a gene-expression signature associated with the survival of HCC patients^18^. The Cancer Genome Atlas (TCGA) program has profiled and analyzed molecular aberrations at the DNA, RNA, protein and epigenetic levels, facilitating the study of the expression of multiple genes simultaneously and offered an opportunity to screen prognostic genes on a global gene scale^19^. The microRNAs (miRNAs), long noncoding RNAs (lncRNAs) and mRNA have been proposed as potential prognostic predictor in HCC^11, 20, 21^. To date, several studies were designed to screen the miRNAs^22–24^ and lncRNAs^25^ related to the clinical outcome of HCC systematically. It is more likely to generate a prognostic signature from mRNAs than miRNAs and lncRNAs because of the considerable number of mRNAs, yet there is no study based on the whole transcriptomics to probe prognostic mRNA in HCC.

TCGA liver hepatocellular carcinoma (LIHC) data sets allow us to correlate transcriptomic profiles to clinical outcomes. In this study, we performed systematic analysis of the whole transcriptomics using Kaplan-Meier survival analysis and revealed the association between the expression of thousands of genes with and survival time of HCC patients. Based on our whole-transcriptome survival analysis, we developed a three-gene prognostic signature consisting of UPB1, SOCS2 and RTN3, and validated this model in two independent microarray data sets retrieved from Gene Expression Omnibus (GEO). Finally, we validated the expression profiles of the outcome predictive genes by western blot and immunohistochemically staining with our HCC samples and performed functional analysis of prognostic genes with TCGA datasheet. To the best of our knowledge, this study is the first to make use of the whole mRNA sequencing data to investigate the cancer specific mRNAs expression patterns and their association with clinical outcome of patients with HCC.

## Results

### The entire workflow of how the signature was generated and validated

**Figure 1:**
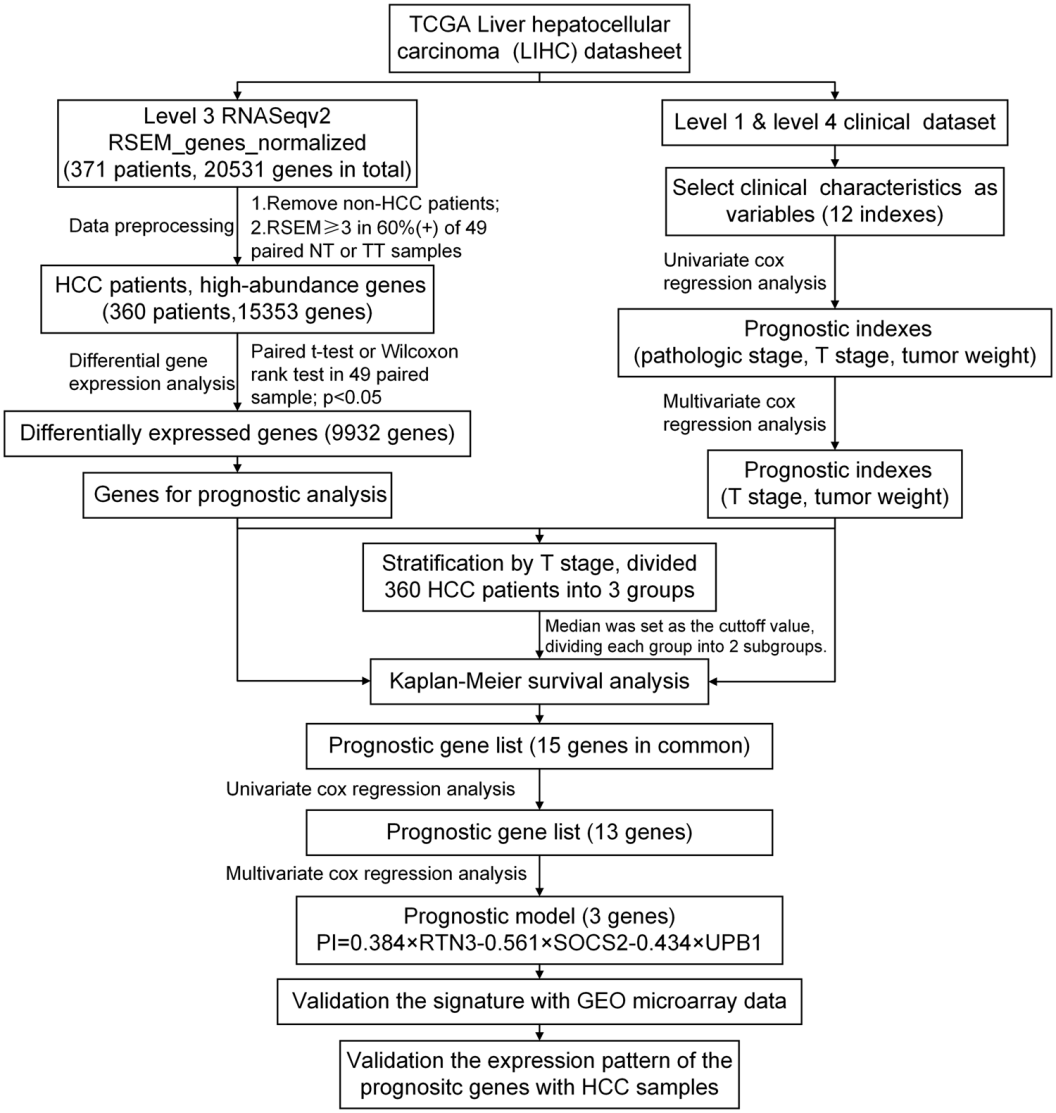
Flowchart describing the process used to generate and validate the prognostic signature in the analysis. The level 3 RNAseqv2 RSEM_genes_normalized files and clinical datasets were downloaded from TCGA. 10 non-HCC patients were ruled out. The gene abundance was filtered by requiring the RSEM normalized count to be ≥3 in ≥60% of 49 paired tumor tissues or nontumor tissues. The remaining 15353 genes were recruited for the differential expression analysis with paired t-test or Wilcoxon signed-rank test. The resulting 9932 differentially expressed genes were candidates for prognostic analysis. Clinical characteristics were selected as variables for univariate and cox regression analysis. T stage and tumor weight were found to be statistically significant in both univariate and multivariate cox regression analysis. 360 patients were stratified by T stage, divided into 3 groups. Kaplan-Meier survival analysis was performed in every group with the median as the cutoff value. We obtained 15 common genes in three groups, and further narrowed this gene list with univariate and cox regression analysis and built a prognostic model that included 3 genes: UPB1, SOCS2 and RTN3. This model was used to calculate risk scores for two independent expression microarrays retrieved from GEO. Finally, we validated the expression pattern of UPB1, SOCS2 and RTN3 by western blot and immunohistochemistry with our HCC samples.

In order to comprehensively analyze the genomic prognostic genes in hepatocellular carcinoma, a workflow outlined the procedure for the analysis was developed (Fig. 1). We acquired the TCGA LIHC level 3 RNA-seq data and clinical datasheet from the TCGA data portal. 10 non-HCC patients and low-abundance genes were filtered, followed by the differential expression analysis. The remaining 360 patients and 9932 differentially expressed genes were enrolled in the prognostic analysis. We divided 360 patients into 3 groups based on T stage, and performed Kaplan-Meier survival analysis in every group individually. Univariate and multivariate cox regression analysis was performed with the 15 common genes, and a three-gene signature was generated. Finally, we validated the signature with microarray data retrieved from GEO (n = 289) and validated the expression pattern of the prognostic genes with our HCC samples (n = 82).

### T stage and tumor weight are independent prognostic clinical parameters in patients with HCC

To rule out the bias caused by the survival-related clinical parameters in the following analysis, we obtained the clinical dataset from TCGA and screened for the survival-related clinical index by univariate and multivariate cox regression analysis (Fig. 1). Univariate cox regression analysis revealed that pathologic stage, T stage, and tumor weight were associated with the survival time (Table 1). Surprisingly, no significant correlation was found between indexes such as Child pugh grade, histologic grade, vascular tumor invasion and clinical outcome according to TCGA clinical dataset. However, only T stage and tumor weight have statistical significance in multivariate cox regression model (Table 1, T stage: hazard ratio [HR] = 1.377; 95% confidence interval [CI] = 1.127–1.683; P = 0.002; tumor weight: hazard ratio [HR] = 3.120; 95% confidence interval [CI] = 2.061–4.724; P < 0.0001), indicating a correlation between tumor burden and survival time.

**Table 1:** The relationship between clinical characteristics and survival time of HCC patients.

Clinical Characteristic	Univariate Analysis		Multivariate Analysis	
	HR (95% CI)	p value	HR (95% CI)	p value
Age	1.012(0.998–1.026)	0.097	—	—
Gender(male,female)	1.204(0.845–1.717)	0.304	—	—
weight	0.995(0.985–1.004)	0.275	—	—
Child pugh grade(a,b,c)	1.529(0.839–2.787)	0.166	—	—
Alcohol consumption(no/yes)	1.029(0.706–1.496)	0.882	—	—
Histologic grade(G1/G2/G3/G4)	1.062(0.841–1.340)	0.615	—	—
Family history(no/yes)	1.166(0.809–1.682)	0.41	—	—
Pathologic stage(1/2/3/4)	1.639(1.339–2.007)	< 0.0001****	—	—
T stage(1/2/3/4)	1.657(1.384–1.985)	< 0.0001****	1.377(1.127–1.683)	0.002**
Vascular tumor invasion(no/yes)	1.358(0.897–2.055)	0.148	—	—
Inflammation(no,mild,severe)	1.141(0.797–1.633)	0.472	—	—
Tumor weight( < median/ > median)	3.513(2.413–5.114)	< 0.0001****	3.120(2.061–4.724)	< 0.0001****

Variables with p < 0.1 in univariate analysis were included in the multivariate analysis using a forward conditional method. Abbreviations: CI, confidence interval; HR, hazard ratio. Statistical significance is marked with the star symbol: **p < 0.01, ****p < 0.0001.

### Identification of 15 common survival-related genes in different T stages

**Figure 2:**
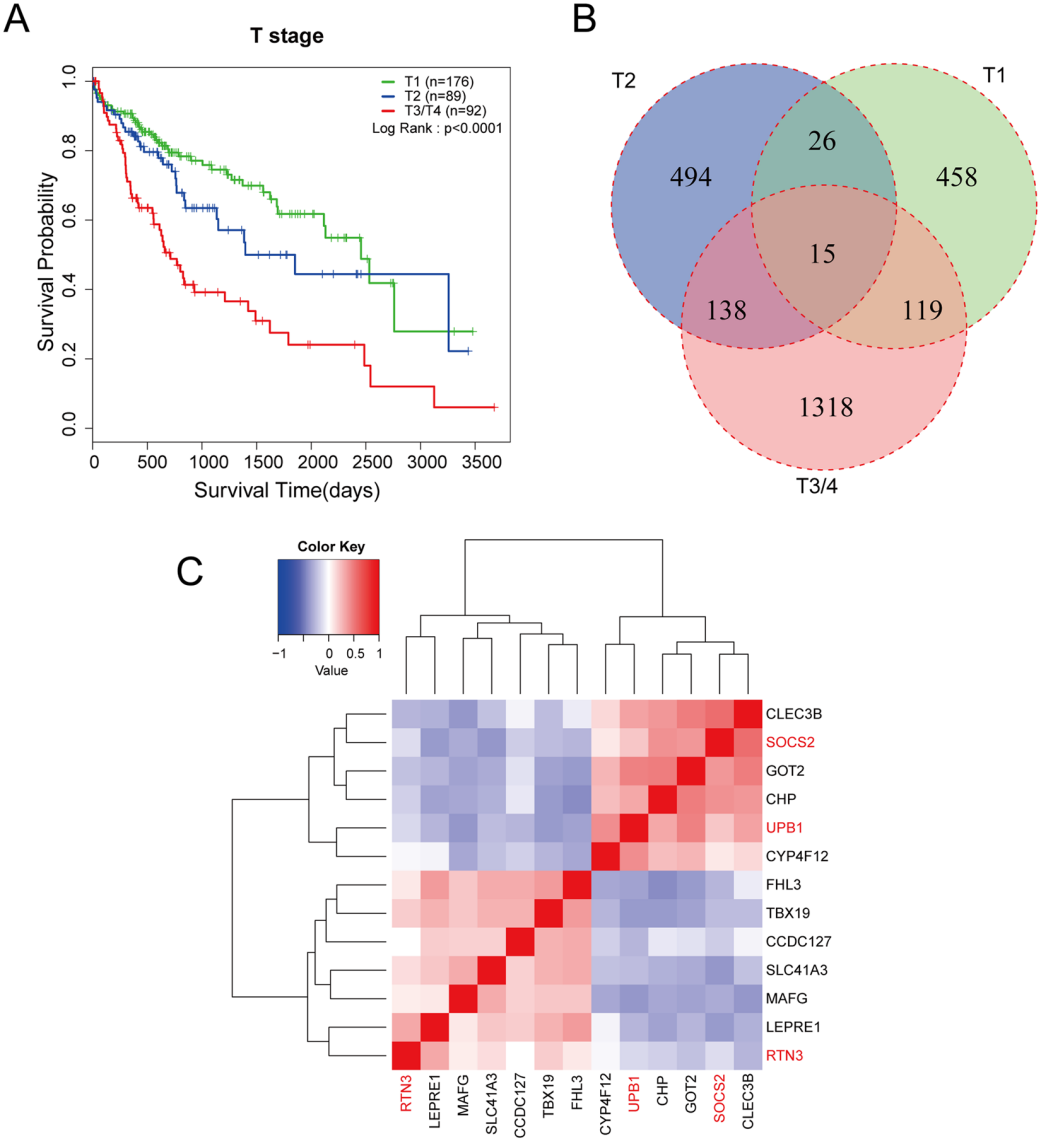
Identification of prognostic genes with T stage stratification. (**A**) Kaplan-Meier curve for overall survival in HCC patients with different T stages from TCGA LIHC dataset. Patients were divided into 3 groups according to the T stages (T1, n = 176; T2, n = 89; T3 and T4, n = 92). Advanced T stage is a significant factor for poor prognosis (p < 0.0001, log-rank test). (**B**) All HCC patients were divided into three groups based on the T stages, and Kaplan-Meier survival analysis was performed in each group separately. Two subgroup were separated on the basis of the median expression level of each gene. 617 survival-related genes were obtained from T1 group, and 672 genes from T2 group, 1590 genes from T3/4 group. 15 common genes were found in different groups. (**C**) Cluster analysis of the 13 survival related genes based on the spearman correlation coefficient. The color in the heatmap represents spearman correlation coefficient. Red indicates positive correlation, and blue indicates negative correlation. 13 genes were clustered into 4 clusters, and RTN3, SOCS2, UPB1 (marked in red) were in different groups.

We then stratified the patients with T stage, resulting in three different groups, with 176 patients in T1 group, 89 patients in T2 group, and 92 patients in T3/4 group. Three patients with unknown T stage were not included in this analysis (Fig. 2A). The median was used as a cutoff value for classification of patients into high and low expression groups. Subsequently, to identify the potential mRNA with prognostic characteristics, we carried out Kaplan–Meier survival analysis of 9932 differentially expressed genes one by one with our R project algorithm in three groups individually, screening for the survival related genes. We obtained an increasing number of candidate genes from T1 to T3/4 group (617 genes in T1 group, 672 genes in T2 group and 1590 genes in T3/4 group, Supplemental Table s1). The overlaps among three groups were illustrated in the Venn diagram (Fig. 2B), and a total of 15 overlapped genes were found in the common region, indicating these 15 genes were associated with survival time regardless of the T stage (Table 2).

**Table 2:** Univariate and multivariate cox regression analysis of the survival-related genes.

Survival-related genes with their IDs	Univariate Analysis		Multivariate Analysis	
	HR (95% CI)	p value	HR (95% CI)	p value
CCDC127.133957	1.420(1.147–1.758)	0.001***	—	—
CHP.11261	0.670(0.538–0.834)	< 0.0001****	—	—
CLEC3B.7123	0.563(0.4500.703)	< 0.0001****	—	—
CYP4F12.66002	0.723(0.583–0.896)	0.003**	—	—
FHL3.2275	1.372(1.112–1.692)	0.003**	—	—
GOT2.2806	0.610(0.489–0.760)	< 0.0001****	—	—
HPR.3250	0.811(0.574–1.146)	0.234	—	—
LEPRE1.64175	1.494(1.202–1.858)	< 0.0001****	—	—
MAFG.4097	1.426(1.149–1.770)	0.01**	—	—
RTN3.10313	1.519(1.220–1.891)	< 0.0001****	1.469(1.180–1.828)	0.001***
SDHAP3.728609	1.050(0.849–1.299)	0.651	—	—
SLC41A3.54946	1.408(1.141–1.737)	0.001***	—	—
SOCS2.8835	0.568(0.453–0.712)	<0.0001****	0.571(0.454–0.717)	<0.0001****
TBX19.9095	1.487(1.197–1.847)	<0.0001****	—	—
UPB1.51733	0.593(0.475–0.740)	<0.0001****	0.648(0.519–0.810)	<0.0001****

Variables with p < 0.1 in univariate analysis were included in the multivariate analysis using a forward conditional method. Gene symbol is in front of the dot, and the numbers behind the dot is the Entrez Gene ID. Abbreviations: CI, confidence interval; HR, hazard ratio. Statistical significance is marked with the star symbol: **p < 0.01, ***p < 0.001, ****p < 0.0001.

### Generation of a three-gene prognostic signature composing of UPB1, SOCS2 and RTN3

To better understand which genes of the 15 candidates are more representative in evaluation of clinical outcome, we further assessed these 15 genes with univariate and multivariate Cox regression analysis in a total of 360 patients. All genes except HPR and SDHAP3 were of statistically significant correlation with survival time in univariate Cox regression analysis (Table 2). Multivariate Cox’s proportional hazards regression analysis indicated that the expression of reticulon 3 (RTN3), suppressor of cytokine signaling 2 (SOCS2) and beta-ureidopropionase 1 (UPB1) were independent predictors for the prognosis of HCC patients (RTN3: hazard ratio [HR] = 1.469; 95% confidence interval [CI] = 0.454–0.717; P = 0.001; SOCS2: hazard ratio [HR] = 0.571; 95% confidence interval [CI] = 2.091–4.911; P < 0.0001; UPB1: hazard ratio [HR] = 0.648; 95% confidence interval [CI] = 0.519–0.810; P < 0.0001; Table 2). Subsequently, based on the estimated Cox regression weights, we established a prognostic model, whose formula was computed as prognostic index (PI) = 0.384 × RTN3 − 0.561 × SOCS2 − 0.434 × UPB1. To explore the relationship among these survival-related genes, we performed cluster analysis based on the Spearman correlation coefficient matrix. Thirteen prognostic genes were classified into four clusters and the genes in the same cluster had similar expression pattern. Three predictive genes obtained from multivariate Cox regression analysis distributed in three different groups (Fig. 2C), which supported the representativeness of the three prognostic genes.

### External validation of the three-gene prognostic model with GEO microarray data

**Figure 3:**
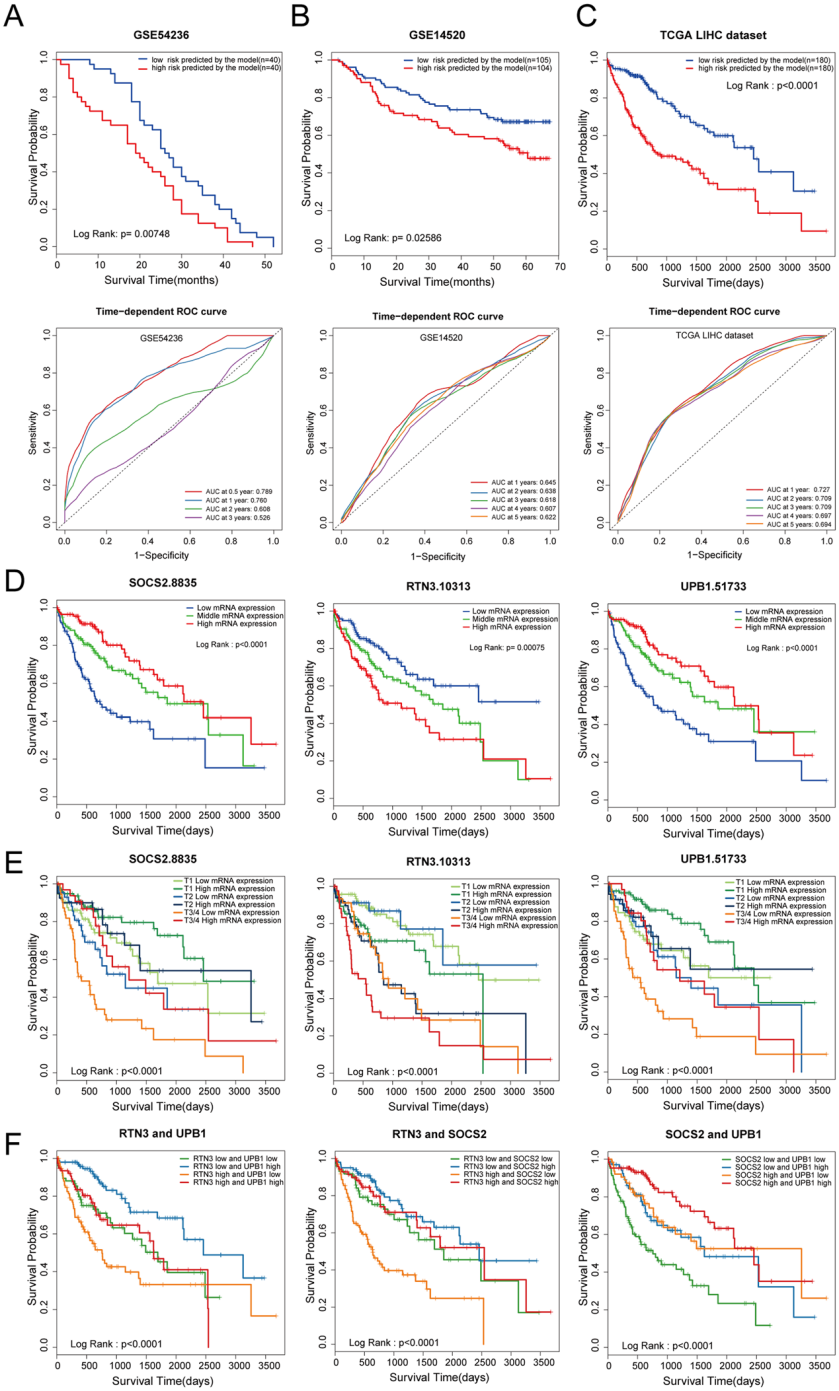
External and internal validation of the three-gene prognostic model. Two independent expression microarray HCC data sets GSE54236 (**A**) and GSE14520 (**B**) were downloaded from the GEO and were used as the signature validation cohort. The risk scores were calculated as Prognostic Index (PI) = 0.384 × RTN3-0.561 × SOCS2-0.434 × UPB1. (**C**) Kaplan-Meier curves of overall survival in the TCGA cohort stratified by the three-gene prognostic signature. We used the time-dependent ROC curve analysis to assessed the prognostic accuracy of the three-gene prognostic signature of the microarray data and LIHC dataset (Fig. 3A lower panel, Fig. 3B lower panel, Fig. 3C lower panel). ROC: receiver operator characteristic. AUC: area under the curve. (**D**) Kaplan–Meier curves of HCC patients based on the SOCS2 (left panel), RTN3 (middle panel), or UPB1 (right panel) expression level. 360 patients were sorted by the RSEM values of a gene, and the lower third of the patients was defined as the low mRNA expression, the upper third as high mRNA expression, thus divided patients into 3 groups. (**E**) The survival curve of HCC patients stratified by T stage and gene expression. Firstly, 360 patients were divided into two groups based on SOCS2 (left panel), RTN3 (middle panel), or UPB1 (right panel) expression. The cutoff value of high and low expression was set as the median. Then, patients were classified into three subgroups according to the T stage. (**F**) Survival curves of 360 HCC patients based on the combination of RTN3 and UPB1 (left panel), RTN3 and SOCS2 (middle panel), SOCS2 and UPB1 (right panel) expression. The cutoff value of high and low expression was set as the median.

To investigate whether our predictive model was appropriate for other datasets, we acquired two independent microarray datasets (GSE14520^26^ and GSE54236^13^) which incorporated gene expression information and survival time of HCC patients from the GEO database. We applied these two datasets to validate our prognostic model respectively. In GSE54236, the survival time for the poor prognosis patients predicted by our model (n = 40) was significantly worse than that of the good prognosis (n = 40) (Fig. 3A upper panel). In GSE14520, the good prognosis group predicted by the signature (n = 105) showed a substantial advantage in overall survival compared to the poor prognosis group (n = 104) (Fig. 3B upper panel). We assessed the prognostic accuracy of the three-gene prognostic signature with time-dependent ROC analysis at varying follow-up times of two validation cohort GSE54236 (Fig. 3A lower panel), GSE14520 (Fig. 3B lower panel) and TCGA LIHC dataset (Fig. 3C lower panel). The area under the curve(AUC) at different cutoff times indicated an acceptable predictive accuracy of the three-gene signature. These results supported the validation of the prognostic signature.

### Internal validation of the three-gene prognostic model with TCGA datasheet

Next, we sought to determine whether the signature composed of UPB1, SOCS2 and RTN3 could be predictive for the clinical outcome of HCC patients. To this end, we tested the model with TCGA LIHC dataset firstly. What stands out in the figure is the difference between the outcome of two groups. Patients with high risk for poor prognosis predicted by the model had a markedly shorter overall survival time compared to patients with low risk, indicated this signature discriminated patients of good prognosis from these of poor outcome effectively (Fig. 3C upper panel).

We subsequently evaluated the prognostic values of UPB1, SOCS2 and RTN3 with Kaplan-Meier survival analysis. The lower thirds of the sorted mRNA expression values were defined as the low expression, and the upper thirds was set as the cutoff point for high expression, thus the total 360 patients were divided into three groups based on the expression value, with 120 patients in each group. The difference among the survival curves of low, middle and high SOCS2 (Fig. 3D left panel), RTN3 (Fig. 3D middle panel) and UPB1 (Fig. 3D right panel) expression groups was statistically significant. As we showed before, T stage is associated with prognosis (Table 1). To ascertain that these three genes distinguishing the survival of HCC patients was not due to a possible correlation with T stage, we depicted the survival curve combined gene expression with T stage. We observed an interesting result when the T stage was taken into consideration. Consistent with the previous result, patients with advanced T stage tended to have poor prognosis overall (Fig. 3E). However, the T1 patients with low SOCS2 expression had a similar survival curve as the T2 patients with high SOCS2 patients. Likewise, patients in T2 stage with low SOCS2 expression had even poorer prognosis than T3 patients with high SOCS2 expression (Fig. 3E left panel), suggesting that SOCS2 is not an insignificant bystander and may play a vital role in the determination of the clinical outcome of HCC patients. Similar results were observed in the survival curve of RTN3 and UPB1 with the combination of T stage (Fig. 3E middle and right panel).

We also evaluated the comprehensive prognostic value of pairwise combination of UPB1, SOCS2 and RTN3. Patient with high RTN3 but low UPB1 expression exhibited shortest survival time, whereas patients with low RTN3 and high UPB1 showed a substantial advantage in overall survival. Patients with low RTN3 and low UPB1 expression or with high RTN3 and high UPB1 expression exhibited median survival time (Fig. 3E, left panel). Similar results could be obtained from the combination of RTN3 and SOCS2 (Fig. 3E, middle panel) or UPB1 and SOCS2 (Fig. 3E, right panel), revealing that pairwise combination of these three genes predicted the survival of HCC patients.

### Validation the expression pattern of UPB1, SOCS2 and RTN3 in HCC samples

**Figure 4:**
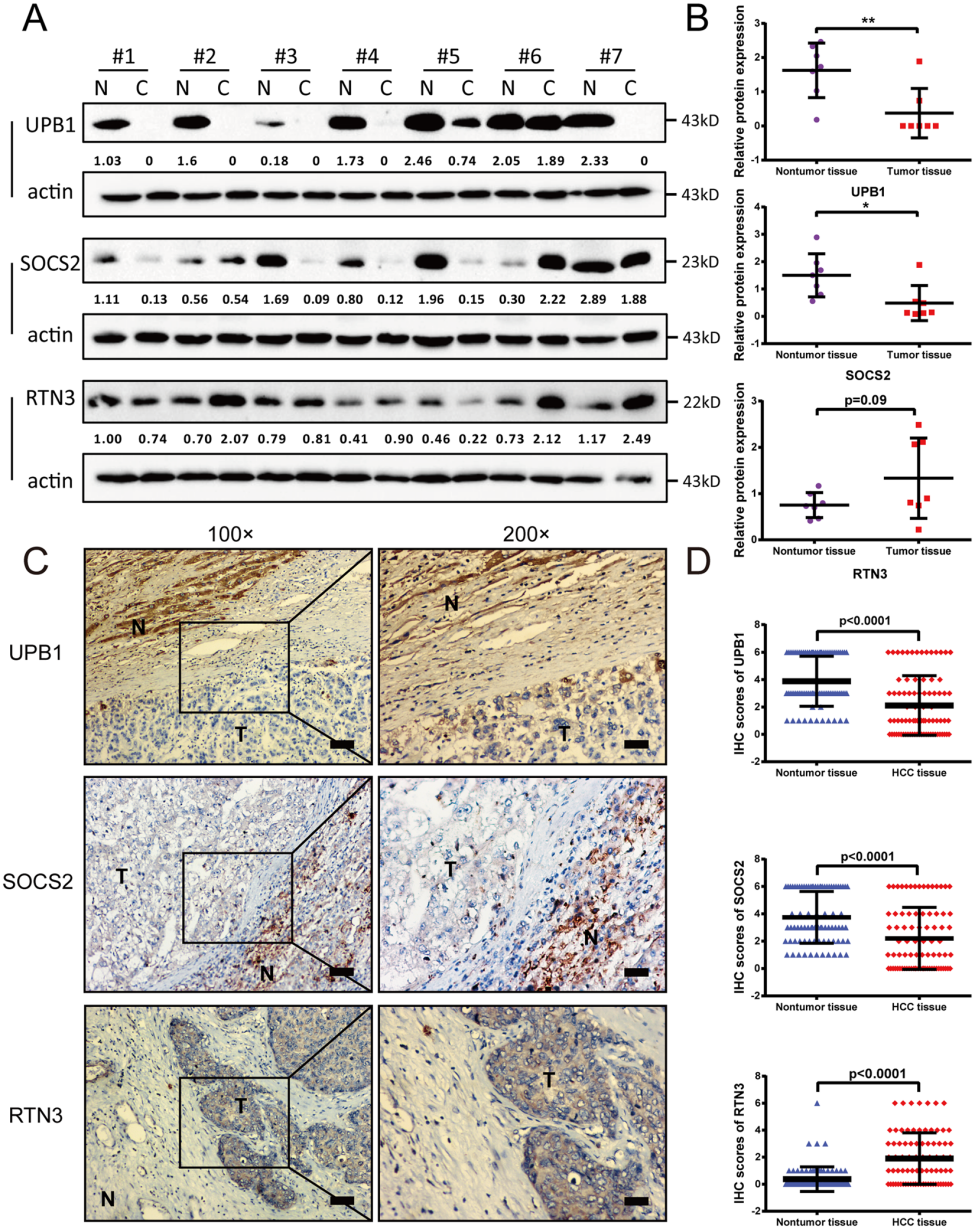
Validation the expression pattern of prognostic genes in HCC samples. (**A**) Western blot analysis of UPB1 (upper panel), SOCS2 (middle panel), and RTN3 (lower panel) in 7 paired normal and tumor tissue samples. (**B**) Western blot quantitative densitometry of the UPB1, SOCS2, and RTN3 relative expressions. Proteins was quantified and normalized to β-actin. Statistical analysis was done using Wilcoxon rank test. Data are expressed as mean values ± SD. *p < 0.05, **p < 0.01. (**C**) Representative images from immunohistochemistry (IHC) staining of UPB1 (upper panel), SOCS2 (middle panel), and RTN3 (lower panel) in 82 normal tissues and paired HCC tissues. Left panel: 100 × magnifications; right panel: 200 × magnifications. Bars = 100 μm. (**D**) Statistical analyses of the average IHC scores of UPB1 (upper panel), SOCS2 (middle panel), and RTN3 (lower panel). Statistical analysis was done using Wilcoxon rank test. Data are expressed as mean values ± SD.

Western blot was employed to examine the expression of UPB1, SOCS2 and RTN3 in 7 pairs of matched HCC samples and adjacent normal tissues. As shown in Fig. 4A and B, UPB1 and SOCS2 exhibited lower expression than the corresponding normal tissues, while RTN3 showed a tendency of increase expression in tumor tissues (p = 0.09). To further confirm the expression pattern of UPB1, SOCS2 and RTN3 in HCC tissues, immunohistochemistry was performed on 82 pairs of HCC and adjacent non-tumorous liver tissues. The baseline clinicopathological characteristics of the HCC patient cohort for IHC was provided in Supplemental Table s3. The results demonstrated that UPB1 and SOCS2 were downregulated in HCC tissues (UPB1: p < 0.0001; SOCS2: p < 0.0001), while the expression of RTN3 was increased in tumor tissues (p < 0.0001) (Fig. 4D). The expression of UPB1 was found to be correlated with tumor capsule invaded, and no other significant association was observed between UPB1, SOCS2 and RTN3 expression and clinicopathological variables perhaps partly because of the relatively small sample size. In addition, the staining pattern indicated that UPB1, SOCS2 and RTN3 were mostly anchored in the cytoplasm (Fig. 4C). Taken together, these data indicated that the expression of SOCS2 and UPB1 was decreased in HCC, while the expression of RTN3 was increased compared with the paired noncancerous tissues.

### Functional analysis of prognostic genes UPB1, SOCS2, and RTN3

**Figure 5:**
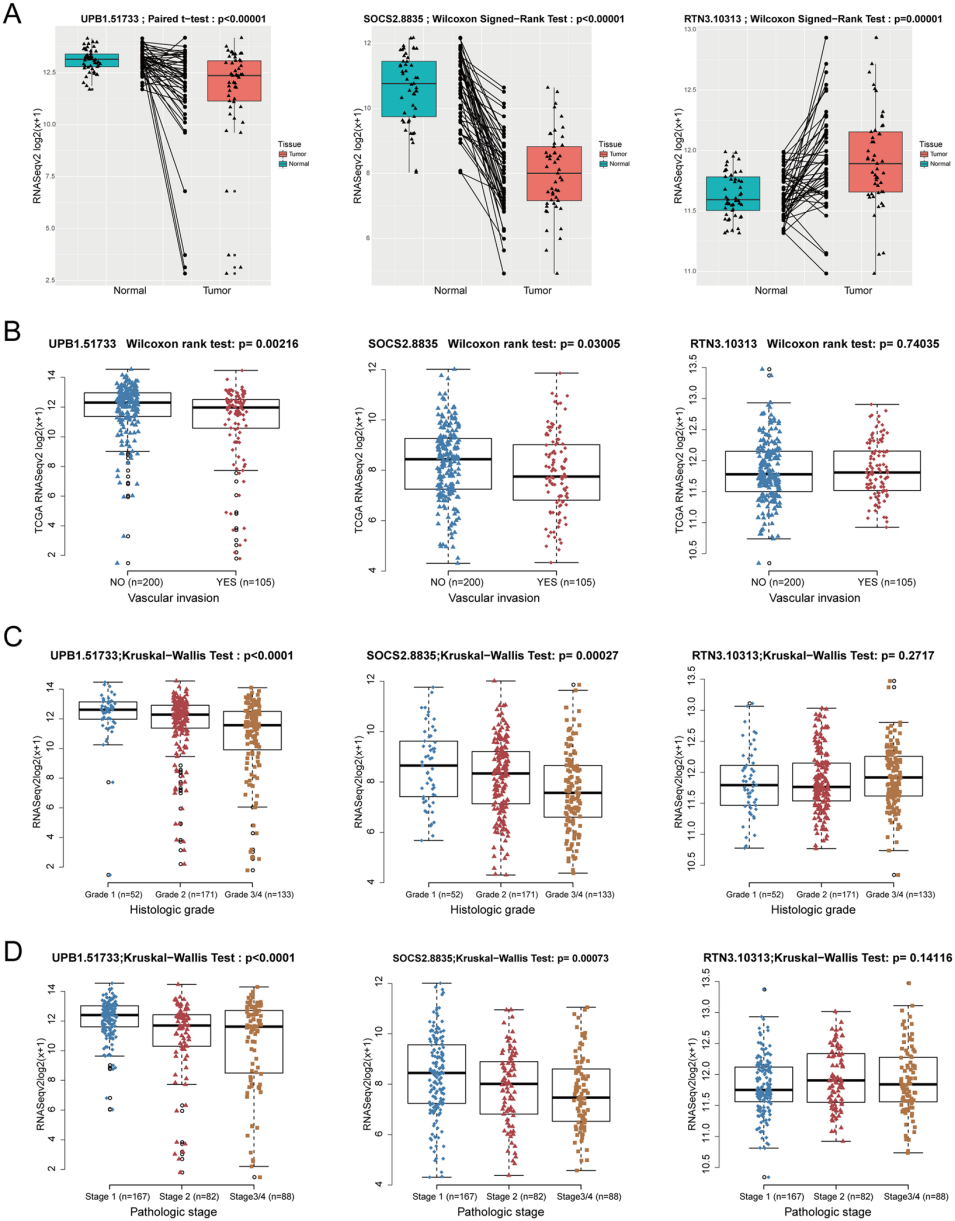
Functional analysis of prognostic genes UPB1, SOCS2, and RTN3 using TCGA dataset. (**A**) The expression pattern of UPB1 (left panel), SOCS2 (middle panel), and RTN3 (right panel) in 49 HCC tissues and paired adjacent non-tumor tissues from TCGA dataset. The Shapiro-Wilk test was applied to determine whether data followed a normal distribution. The paired t-test was applied to normally distributed data otherwise the Wilcoxon rank test for paired data was applied to assess the expression pattern in HCC tissues. UPB1 and SOCS2 were downregulated in HCC tissues, while RTN3 was upregulated. (**B**) Association of UPB1 (left panel), SOCS2 (middle panel), and RTN3 (right panel) expression with vascular invasion. Mann-Whitney-Wilcoxon test showed significant differences between vascular invasion group (n = 200) and no vascular invasion group (n = 105) of UPB1 and SOCS2 expression, but not RTN3. (**C**) Association of UPB1 (left panel), SOCS2 (middle panel), and RTN3 (right panel) expression with the histologic grade. 356 patients with histologic grade information (grade 1, n = 52; grade 2, n = 171; grade 3/4, n = 133) were recruited for the analysis. The Kruskal-Wallis test revealed a negative correlation between UPB1 and SOCS2 expression and histologic grade, while no significant differences was found in RTN3 expression in different groups. (**D**) Association of UPB1 (left panel), SOCS2 (middle panel), and RTN3 (right panel) expression with pathologic stage. 337 patients with pathologic grade information (stage 1, n = 167; stage 2, n = 82; stage 3/4, n = 88) were recruited for the analysis. The Kruskal-Wallis test was performed. UPB1 and SOCS2 were found to be associated with pathologic stage, while RTN3 was not.

Comparing tumor tissue with the surrounding nontumor tissue is usually the first step in search for biomarkers, aimed to identify biological characteristics unique to tumor tissue [19]. To this end, we performed differential expression analysis in 49 paired tissues at the beginning of the study and obtained 9932 differentially expressed genes (Fig. 1). RTN3 was upregulated in HCC tumor tissue compared to nontumor tissue (p = 0.00001, Fig. 5A right panel), while expression of UPB1 in tumor tissue was significantly lower than that in nontumor tissue (p < 0.00001, Fig. 5A left panel). SOCS2 was also downregulated in tumor tissues (p < 0.00001). Besides, the expression of SOCS2 was observed to be downregulated in every pair of tissues (Fig. 5A middle panel).

We investigated the potential interactions among clinicopathological variables and UPB1, SOCS2 and RTN3 expression. As shown in Supplemental Table s2, UPB1 expression (between low expression group and high expression group) was closely correlated with tumor weight (p < 0.0001), histologic grade (p < 0.0001), pathological stage (p < 0.0001), T stage (p < 0.0001) and survival status (p < 0.0001). The expression of SOCS2 (between low expression group and high expression group) was correlated with histologic grade (p < 0.0001), family history (p  = 0.046), pathological stage (p = 0.001), T stage (p =  0.001), vascular invasion (p = 0.034) and vital status (p < 0.0001). The expression of RTN3 (between low expression group and high expression group) was negatively correlated with the vital status. No significant associations were observed between RTN3 expression and other clinicopathological variables in TCGA LIHC dataset.

Additionally, we evaluated the expression pattern of UPB1, SOCS2 and RTN3 in patients with or without vascular invasion. The expression of UPB1 (p = 0.002) and SOCS2 (p = 0.03) was significantly lower in HCC patients with vascular invasion (n = 105), compared with patients without vascular invasion (n = 200), while no significant differences were found in RTN3 expression (Fig. 5B). Furthermore, we investigated the expression profiles of the three predictive genes with histologic grade (Fig. 5C) and pathologic stage (Fig. 5D). Notably, with the advance of histologic grade and pathologic stage, the expression of UPB1 and SOCS2 were downregulated gradually. No significant differences among different groups were evident of RTN3 expression. These results indicated that these three predictive genes, especially SOCS2 and UPB1 were associated with tumor progression in HCC.

## Discussion

HCC is one of the deadliest solid malignancies, because recurrence occurs in most patients even after undergoing potentially curative treatment. It’s important to predict clinical outcome and adopt novel interventional measures in time for the patients at high risk for recurrence. Recent progress in integrated “Omics” analysis shed new light on HCC biomarker screening and validation. In this study, we investigated the prognostic clinicopathological characteristics and the predictive genes based on RNA-seq data from the public databases, established and validated of a three-gene prognostic signature containing UPB1, SOCS2 and RTN3.

Recent “Omics” technologies have opened the door to screening biomarkers in a high throughput manner. Microarray and the next generation sequencing technologies have facilitated the study of the expression of multiple genes simultaneously, offering an opportunity to probe prognostic genes on a global genome scale. However, reproducibility of the data is an inevitable problem, especially when the data were generated from different technologies and platforms. One well-known study that was often cited in research on prognostic biomarker screening for HCC is that of Hoshida (2008), which failed to identify an outcome associated signature based on gene-expression profiles of tumor tissue, but discovered and validated a gene-expression signature associated with survival based on the profiles of the surrounding nontumoral liver tissue^18^. However, we succeeded in developing a three-gene signature including UPB1, SOCS2 and RTN3 based on gene-expression profiles of tumor tissue in this study, and fortunately we have validated this model with two independent GEO microarray datasets produced from different platforms. There are several possible explanations for the inconsistency. Firstly, Hoshida’s study was accomplished by hybridization-based techniques (microarrays), and only 6000 oligonucleotide probes were included in the analyses, while TCGA datasets were generated by the next-generation sequencing technologies which do not use molecular hybridization to capture transcripts and therefore do not depend on the probe numbers. The limitation of the cross-platform analysis and the limited number of probes of Hoshida’s study might partly explained the inconsistency of the two studies. The second possible explanation for the discrepancy was the heterogeneity of HCC patients. The tissue samples of 80 HCC patients in the training set in Hoshida’s study were all from a single center in Japan with high hepatitis virus infection rate (HBV: 23.75%, HCV:72.5%), while the patients in TCGA LIHC dataset were from multicenter consisting of different races including Asian, White and Black with low viral hepatitis rate (Supplemental Table s2). Another point which is easy to be neglected but could have an impact is the tissue procurement procedures. The intra- and post-operative cold and warm ischemia were reported to impact the RNA^27^, protein^28^ and metabolites^29^ of clinical tissue specimens. The tissue procurement procedures can have a significant impact on the quality of specimens and might influence the reproducibility of analytical data sets for biomarker. Because we chose death to be the clinical outcome of our study, whether the death of patients was caused by HCC would influence the accuracy of our results.

The role of RTN3 in Alzheimer’s disease (AD) has been widely investigated. Transgenic mice overexpressing RTN3 developed neuritic abnormalities^30, 31^. Additionally, RTN3 is reported to interact directly with a viral nonstructural protein and is important for viral replication^32^. However, RTN3 was found to limit the replication of HCV^33^. It has been observed that RTN3 was overexpressed in astrocytoma compared to non-cancerous tissue^34^. It’s reported that RTN3 could interact with the oncogene Ras at endoplasmic reticulum^35^. Further work needs to be done to assess the role of RTN3 in tumorigenesis especially in HCC.

SOCS2 was reported to negatively regulate the growth hormone signaling *in vitro* and *in vivo* ^36^ and was identified as an important regulator of hepatic homeostasis in response to high-fat dietary stress^37^. In addition, SOCS2 was a crucial intracellular mediator of the anti-inflammatory actions. SOCS2 deficient mice had uncontrolled production of proinflammatory cytokines upon infection with an intracellular pathogen^38^. Livers from high-fat-diet-fed SOCS2^−/−^ mice showed increased Nuclear Factor κB (NF-κB) activity as well as elevated expression of genes for the inflammatory cytokines Interferon (IFN)-γ and Interleukin (IL)-6^37^. Over the last years, SOCS2 has emerged as potential tumor suppressor-like gene in several tumors. Previous studied have revealed that SOCS2 was downregulated in ovarian, breast carcinomas, pulmonary adenocarcinoma and HCC^39–42^. Consistent with our study, it has been suggested that SOCS2 was an independent predictor for survival of patients with breast cancer^40^, colorectal cancer^43^, and HCC^42^, and patients with low expression of SOCS2 displayed shorter survival time. SOCS2 deficiency promotes spontaneous intestinal tumorigenesis in a specific mice model driven by mutations in the APC/beta-catenin pathway^44^. Although conflicting results were reported that SOCS2 expression was upregulated in prostate cancer^45, 46^, these findings provide insights into the role of SOCS2 in tumor suppression and highlight the potential usefulness of SOCS2 as a prognostic prediction biomarker.

UPB1 catalyzes the last step in the pyrimidine degradation pathway. Thus far, few studies have explored the relationship between UPB1 and cancer. In a study aimed to discovery novel circulation biomarkers for HCC by integrating various bioinformatics tools, they identified a seven-protein predictive signature including UPB1^47^. Further experimental investigations are needed to estimate the function of UPB1 in tumors.

The initial aim of our study was to seek out several prognostic genes from the RNA-seq data, and validate the expression pattern and the prognostic value in protein level and then establish a predictive immunohistochemical staining signature and finally apply to clinical research and practice because of the easy accessibility of immunohistochemistry to patients. Though we validated the expression pattern of UPB1, SOCS2 and RTN3 by western blot and immunohistochemistry, we didn’t associate the expression of these genes to the survival of patients, because the subsequent validation process is difficult and costly. We acquired three survival-related genes and it was reported that these three genes played important roles in tumors. However, further research should be undertaken to investigate the function of UPB1, SOCS2 and RTN3 in HCC. The three-gene signature is promising, but the patient cohorts from both TCGA and GEO were retrospective, and prospective cohorts were needed to validate this signature. Although some limitations remain in our study, we have performed the first whole mRNA transcriptome survival analysis in HCC to search for predictive biomarkers, and we think our method can be extended to other cancer type.

In conclusion, we identified a three-gene predictive signature comprised of UPB1, SOCS2 and RTN3 for hepatocellular carcinoma and validated these three genes expression pattern in HCC tissues. This study may contribute to provide significant clinical implications for the prognosis prediction of HCC patients. However, what remains unclear is how these genes impact survival and it is beyond the scope of this study. Further investigation and experimentation are needed to elucidate the biological mechanisms of UPB1, SOCS2 and RTN3 in HCC development and progression.

## Materials and Methods

### Experimental protocol approval

All experimental protocols were approved by the research ethics committee of Drum Tower hospital and research ethics approval for this project was granted from the same institution. Utilization of data was conducted in accordance with TCGA and GEO data access policies. All analyses were performed in accordance with relevant guidelines and regulations.

### Data sources and processing

TCGA LIHC level 3 RNAseqv2 data (RSEM_genes_normalized file) and clinical datasheet were obtained from the TCGA data portal (up to Jan 28, 2016). 371 patients containing 49 patients with paired normal liver tissues and 20531 genes were included. 10 patients whose pathological diagnoses were not hepatocellular carcinoma were ruled out. The gene abundance was filtered by requiring the RSEM normalized count to be ≥3 present in no less than 60% samples of the 49-paired tumor or normal tissues. The log2 transformation of RSEM was adopted in all plots. Two independent expression microarray HCC data sets (GSE14520 and GSE54236) were downloaded from the Gene Expression Omnibus (GEO) database (http://www.ncbi.nlm.nih.gov/geo/), in which outcome and survival time of the patients were incorporated. The survival time of HCC patients in microarrays was downloaded from ArrayExpress (http://www.ebi.ac.uk/arrayexpress/). GSE14520 (based on GPL3921 platform) and GSE54236 (based on GPL6480) enrolled 209 and 80 HCC patients with survival time, respectively.

### Differential gene expression analysis

The differential expression gene (DEG) analysis was performed on 49 paired HCC samples. The Shapiro-Wilk test was applied to determine whether the difference of normal/tumor RSEM followed a normal distribution. The paired t-test was applied to normally distributed data otherwise the Wilcoxon rank test for paired data was applied to assess whether a gene was differentially expressed between tumor and normal samples. Statistical significance was determined using p < 0.05.

### Survival analysis

For survival analysis, we used the Kaplan-Meier method to analyze the correlation between overall survival time and gene expression. The statistical significances of overall survival were determined using the Log-Rank test. Survival analysis was performed in R (version 3.3.1) and the survival curve was generated by R (version 3.3.1). The median was used as a cutoff value for classification of patients into high and low expression groups. Likewise, we use two tertiles to split patients into three groups with each gene expression, trisected patients into low expression group, middle expression group, and high expression group. We used R software version 3.3.1 and the “survivalROC” package to do the time-dependent ROC curve analysis.

### Signature development

Prognostic index model was created using the three-gene signature based on multivariate Cox survival analysis. This algorithm is based on an importance score assigned to each gene. Using the Cox regression models, we calculated a risk score for each patient based on their individual expression levels of the three genes, where prognostic index (PI) = (0.384 × relative expression value of RTN3) − (0.561 × relative expression value of SOCS2) − (0.434 × relative expression value of UPB1). The median expression was used as a cutoff value, and the relative expression value was defined as follows: score 1, <median; score 2, >median.

### Human tissue samples

Liver tissues were collected from patients undergoing hepatectomy with confirmed diagnosis of HCC at Department of Hepatobiliary Surgery of the Affliated Drum Tower Hospital of Nanjing University Medical School. Written informed consent was obtained from all patients or their guardians for the use of the biospecimens for research purposes. The samples were frozen in liquid nitrogen immediately after surgical resection and stored at −80 °C until further analysis.

### Western blot

Cells were lysed in RIPA buffer containing a protease inhibitor cocktail (Roche, Mannheim, Germany, 11873580001). Protein concentration was determined. Equal amounts (20 ug/lane) of proteins were separated by sodium dodecyl sulfate - polyacrylamide gel electrophoresis (SDS-PAGE) and transferred to a polyvinylidene fluoride (PVDF) membrane (Roche, 03010040001). After blocking with 5% nonfat milk in Tris-buffered saline for 1 h, the membrane was incubated overnight at 4 °C with specific primary antibodies, followed by incubation with appropriate HRP conjugated secondary antibodies. Signals were detected using an enhanced chemiluminescence reagent (Millipore, Darmstadt, Germany, WBKLS0500) and subjected to Alpha Innotech Flour Chem-FC2 imaging system (Alpha Innotech, San Leanardo, CA). The antibodies used in WB were as follows: anti-RTN3 (Abcam, ab68328, 1:1000 dilution), anti-SOCS2 (Abcam, ab3692, 1:1000 dilution), anti-UPB1 (Abcam, ab157195, 1:1000 dilution), anti-β-Actin (Cell Signaling Technology, #4970, 1:1000 dilution), horseradish peroxidase (HRP)-conjugated secondary antibodies (Multisciences, GAR007 and GAM007, 1:2000 dilution). Quantitative densitometry was performed using Photoshop CC (Adobe, CA, USA).

### Immunohistochemistry

Formalin-fixed and paraffin-embedded HCC sections with a thickness of 4 μm were dewaxed in xylene and graded alcohols, hydrated and washed in phosphate buffered saline (PBS). Antigen retrieval was done by heat treatment of the deparaffinized sections in a pressure cooker in EDTA-TRIS (PH9.0) for 2 minutes. After the initial processing steps, sections were incubated overnight with primary antibody at 4 °C. The primary antibodies used in IHC were as follows: anti-RTN3 (Sigma, # HPA015649, 1:300 dilution), anti-SOCS2 (Abcam, ab74533, 1:150 dilution), anti-UPB1 (Abcam, ab157195, 1:600 dilution). A subsequent reaction was performed with biotin-free HRP enzyme-labeled polymer from an EnVision plus detection system (DAKO, K5007, Glostrup, Denmark). Positive reactions were visualized with diaminobenzidine (DAB) solution followed by counterstaining with hematoxylin. Negative controls were performed using non-immune goat serum instead of the primary antibodies. The protein expression was assessed by a certified pathologist who was blind to all clinical and biological variables using a semi-quantitative scoring consisting of an assessment of both staining intensity (scale 0–3: 0, none; 1, mild; 2, moderate; and 3, intense.) and the percentage of positive cells (0–4: 0, <5% cells; 1, 6–25% cells; 2, 25–50% cells; 3, 51–75% cells; and 4, >75% cells), yielding an overall score.

### Statistics

Univariate and multivariate survival analysis were performed using the Cox proportional hazards regression model. Only variables with p < 0.1 on univariate analysis were incorporated into the multivariate Cox regression analysis. The paired t-test or Wilcoxon signed-rank test were used for paired samples to make a statistical comparison between groups. The Kruskal-Wallis test and Mann-Whitney-Wilcoxon test were used for ranked data as appropriate. All testing was carried out using Prism 6.0 (GraphPad, San Diego, USA) or SPSS (version 23.0; Chicago, USA) or R (version 3.3.1, Auckland, NZ). The two-sided p value less than 0.05 was defined as statistically significant for all statistical analyses. The data were plotted as mean ± standard deviation (SD).

## Electronic supplementary material

Supplemental Table s1

Supplementary Info 2

## References

[1] Torre LA (2015). Global cancer statistics, 2012. CA: a cancer journal for clinicians.

[2] Ferlay J (2015). Cancer incidence and mortality worldwide: sources, methods and major patterns in GLOBOCAN 2012. Int J Cancer.

[3] Bruix J, Reig M, Sherman M (2016). Evidence-Based Diagnosis, Staging, and Treatment of Patients With Hepatocellular Carcinoma. Gastroenterology.

[4] Yu SJ (2016). A concise review of updated guidelines regarding the management of hepatocellular carcinoma around the world: 2010–2016. Clinical and molecular hepatology.

[5] Diaz-Gonzalez A, Forner A (2016). Surveillance for hepatocellular carcinoma. *Best practice & research*. Clinical gastroenterology.

[6] Bruix J, Gores GJ, Mazzaferro V (2014). Hepatocellular carcinoma: clinical frontiers and perspectives. Gut.

[7] Zheng, J. *et al*. Actual 10-Year Survivors After Resection of Hepatocellular Carcinoma. *Ann Surg Oncol*, 1–9, doi:10.1245/s10434-016-5713-2 (2016). 10.1245/s10434-016-5713-2 PMC5609806 27921192

[8] Hanazaki K, Kajikawa S, Koide N, Adachi W, Amano J (2001). Prognostic factors after hepatic resection for hepatocellular carcinoma with hepatitis C viral infection: univariate and multivariate analysis. Am J Gastroenterol.

[9] Ijichi M (2002). alpha-Fetoprotein mRNA in the circulation as a predictor of postsurgical recurrence of hepatocellular carcinoma: a prospective study. Hepatology.

[10] Tangkijvanich P (2000). Clinical characteristics and prognosis of hepatocellular carcinoma: analysis based on serum alpha-fetoprotein levels. J Clin Gastroenterol.

[11] Singhal A, Jayaraman M, Dhanasekaran DN, Kohli V (2012). Molecular and serum markers in hepatocellular carcinoma: predictive tools for prognosis and recurrence. Crit Rev Oncol Hematol.

[12] Mann CD (2007). Prognostic molecular markers in hepatocellular carcinoma: a systematic review. Eur J Cancer.

[13] Villa E (2016). Neoangiogenesis-related genes are hallmarks of fast-growing hepatocellular carcinomas and worst survival. Results from a prospective study. Gut.

[14] Zhou L, Liu J, Luo F (2006). Serum tumor markers for detection of hepatocellular carcinoma. World journal of gastroenterology: WJG.

[15] Yu DC, Chen J, Ding YT (2010). Hypoxic and Highly Angiogenic Non-Tumor Tissues Surrounding Hepatocellular Carcinoma: The ‘Niche’ of Endothelial Progenitor Cells. International Journal of Molecular Sciences.

[16] Yu D (2007). Particular distribution and expression pattern of endoglin (CD105) in the liver of patients with hepatocellular carcinoma. BMC Cancer.

[17] Yu D (2015). Kidney-type glutaminase (GLS1) is a biomarker for pathologic diagnosis and prognosis of hepatocellular carcinoma. Oncotarget.

[18] Hoshida Y (2008). Gene expression in fixed tissues and outcome in hepatocellular carcinoma. The New England journal of medicine.

[19] Cancer Genome Atlas Research N (2013). The Cancer Genome Atlas Pan-Cancer analysis project. Nat Genet.

[20] Li C (2015). Progress and Prospects of Long Noncoding RNAs (lncRNAs) in Hepatocellular Carcinoma. Cellular physiology and biochemistry: international journal of experimental cellular physiology, biochemistry, and pharmacology.

[21] He S, Zhang DC, Wei C (2015). MicroRNAs as biomarkers for hepatocellular carcinoma diagnosis and prognosis. Clinics and research in hepatology and gastroenterology.

[22] Shi KQ (2015). Hepatocellular carcinoma associated microRNA expression signature: integrated bioinformatics analysis, experimental validation and clinical significance. Oncotarget.

[23] Zhang J, Chong CC, Chen GG, Lai PB (2015). A Seven-microRNA Expression Signature Predicts Survival in Hepatocellular Carcinoma. PLoS One.

[24] Lu M (2017). A novel microRNAs expression signature for hepatocellular carcinoma diagnosis and prognosis. Oncotarget.

[25] Zhang J, Fan D, Jian Z, Chen GG, Lai PB (2015). Cancer Specific Long Noncoding RNAs Show Differential Expression Patterns and Competing Endogenous RNA Potential in Hepatocellular Carcinoma. PLoS One.

[26] Roessler S (2010). A unique metastasis gene signature enables prediction of tumor relapse in early-stage hepatocellular carcinoma patients. Cancer Res.

[27] Kap M (2015). The influence of tissue procurement procedures on RNA integrity, gene expression, and morphology in porcine and human liver tissue. Biopreservation and biobanking.

[28] Gundisch S (2012). Variability of protein and phosphoprotein levels in clinical tissue specimens during the preanalytical phase. J Proteome Res.

[29] Cacciatore S (2013). Effects of intra- and post-operative ischemia on the metabolic profile of clinical liver tissue specimens monitored by NMR. J Proteome Res.

[30] Hu X (2007). Transgenic mice overexpressing reticulon 3 develop neuritic abnormalities. The EMBO journal.

[31] Araki W (2013). Reduction of beta-amyloid accumulation by reticulon 3 in transgenic mice. Current Alzheimer research.

[32] Tang WF (2007). Reticulon 3 binds the 2C protein of enterovirus 71 and is required for viral replication. The Journal of biological chemistry.

[33] Wu MJ, Ke PY, Hsu JT, Yeh CT, Horng JT (2014). Reticulon 3 interacts with NS4B of the hepatitis C virus and negatively regulates viral replication by disrupting NS4B self-interaction. Cellular microbiology.

[34] Huang X (2004). Overexpression of human reticulon 3 (hRTN3) in astrocytoma. Clinical neuropathology.

[35] Su Y (2007). Selectively oncolytic mutant of HSV-1 lyses HeLa cells mediated by Ras/RTN3. Cancer Biol Ther.

[36] Greenhalgh CJ (2005). SOCS2 negatively regulates growth hormone action *in vitro* and *in vivo*. J Clin Invest.

[37] Zadjali F (2012). SOCS2 deletion protects against hepatic steatosis but worsens insulin resistance in high-fat-diet-fed mice. FASEB journal: official publication of the Federation of American Societies for Experimental Biology.

[38] Machado FS (2006). Anti-inflammatory actions of lipoxin A4 and aspirin-triggered lipoxin are SOCS-2 dependent. Nat Med.

[39] Sutherland KD (2004). Differential hypermethylation of SOCS genes in ovarian and breast carcinomas. Oncogene.

[40] Haffner MC (2007). Favorable prognostic value of SOCS2 and IGF-I in breast cancer. BMC Cancer.

[41] Wikman H (2002). Identification of differentially expressed genes in pulmonary adenocarcinoma by using cDNA array. Oncogene.

[42] Qiu X (2013). Reduced expression of SOCS2 and SOCS6 in hepatocellular carcinoma correlates with aggressive tumor progression and poor prognosis. Mol Cell Biochem.

[43] Letellier E (2014). Identification of SOCS2 and SOCS6 as biomarkers in human colorectal cancer. Br J Cancer.

[44] Newton VA (2010). Suppressor of cytokine signaling-2 gene disruption promotes Apc(Min/+) tumorigenesis and activator protein-1 activation. The American journal of pathology.

[45] Hoefer J (2014). SOCS2 correlates with malignancy and exerts growth-promoting effects in prostate cancer. Endocr Relat Cancer.

[46] Zhu JG (2013). Expression of SOCSs in human prostate cancer and their association in prognosis. Mol Cell Biochem.

[47] Awan FM (2015). Identification of Circulating Biomarker Candidates for Hepatocellular Carcinoma (HCC): An Integrated Prioritization Approach. PLoS One.

