# Supplementary material for: Development and Validation of a Three-gene Prognostic Signature for Patients with Hepatocellular Carcinoma
Source: Sci Rep. 2017 Jul 17;7:5517. doi: 10.1038/s41598-017-04811-5 (PMC5514049; doi:10.1038/s41598-017-04811-5)
Supplement: Supplementary file 2 — Supplementary Info 2 [file 41598_2017_4811_MOESM2_ESM.pdf]

Development and Validation of a Three-gene Prognostic Signature for Patients with Hepatocellular Carcinoma

Binghua Li[1,*], Wendu Feng[1,*], Ouyang Luo[1], Tiancheng Xu[1], Yajuan Cao[1], Hongyan Wu[2], Decai Yu[1]& Yitao Ding[1]

[1]Department of Hepatobiliary Surgery, the Affliated Drum Tower Hospital, Medical School of Nanjing University, Nanjing, 210093, China
[2]Department of Pathology, the Affliated Drum Tower Hospital, Medical School of Nanjing University, Nanjing, 210093, China
[*]These authors have contributed equally to this work. Correspondence and requests for materials should be addressed to D.C.Y. (email: dryudecai@qq.com) or Y.T.D. (email: drdingyitao@sina.com.cn)

Supplemental Table s2

Baseline clinicopathological characteristics and relationship between UPB1, SOCS2 and RTN3 expression and clinicopathological parameters of HCC patients from TCGA dataset. Clinical datasheet was downloaded from the TCGA data portal and the characteristics were grouped by UPB1, SOCS2 and RTN3 expression. Patients with unavailable clinicopathological variables were not included in the analysis. The median was used as cutoff value for classification of patients into high or low expression groups. The count data were analyzed with Pearson Chi-Square or Fisher's Exact Test. The quantitative data were analyzed with t-test or Mann-Whitney U test and the ordinal data were analyzed with nonparametric statistics as appropriate. Significance level: $p<0.05$.

Characteristics	Patients	UPB1 expression		p value	SOCS2 expression		p value	RTN3 expression		p value
		Low	High		Low	High		Low	High	
Age				0.409			0.998			0.14
Mean	59.6	58.79	60.4		59.56	59.64		60.31	58.88	
SD	13.3	13.56	12.96		13.31	13.26		13.58	12.95	
Gender				0.574			0.736			0.431
Male	243(67.5%)	119	124		120	123		118	125	
Female	117(32.5%)	61	56		60	57		62	55	
Weight				0.023			0.313			0.779
Mean	72.8	70.99	74.61		71.97	73.67		72.44	73.22	
SD	19.6	19.8	19.32		19.58	19.67		18.17	20.99	
Race										
Asian	155(43.1%)	78	77	0.522	84	71	0.496	71	84	0.264
White	176(48.9%)	87	89		84	82		95	81	
Other	19(5.3%)	12	7		10	9		8	11	
Child pugh grade				0.883			0.511			0.261
a	211(90.6%)	92	119		99	112		107	104	
b	21(9.0%)	10	11		12	9		14	7	
c	1(0.4%)	0	1		0	1		0	1	
Tumor weight				0.0001			0.337			0.843
Mean	271.5	330.89	212.17		271.5	271.56		286.89	256.17	
SD	346.5	386.11	290.95		338.25	355.53		386.14	303.06	
Vital status				0.001			0.0001			0.006
Alive	231(64.2%)	101	130		98	133		128	103	
Dead	129(35.8%)	79	50		82	47		52	77	
Alcohol consumption				0.194			0.402			0.493
No	226(66.3%)	107	119		111	115		115	111	
Yes	115(33.7%)	63	52		62	53		54	61	
Viral hepatitis				0.44			0.144			0.815
No	230(67.4%)	118	112		123	107		115	115	
Yes	111(32.6%)	52	59		50	61		54	57	
Histologic grade				0.0001			0.0001			0.091

G1	53(14.9%)	19	34		21	32		27	26	
G2	171(48.2%)	75	96		75	96		93	78	
G3	120(33.8%)	77	43		74	46		55	65	
G4	11(3.1%)	7	4		8	3		2	9	
Family history				0.07			0.046			0.48
No	202(65.0%)	107	95		111	91		99	103	
Yes	109(35.0%)	46	63		47	62		58	51	
Pathological stage				0.0001			0.001			0.165
Stage I	167(49.7%)	66	101		70	97		91	76	
Stage II	81(24.1%)	46	35		42	39		35	46	
Stage III	84(25.0%)	52	32		55	29		41	43	
Stage IV	4(1.2%)	3	1		2	2		1	3	
T stage				0.0001			0.001			0.22
T1	176(49.3%)	69	107		75	101		96	80	
T2	89(24.9%)	52	37		46	43		36	53	
T3	79(22.1%)	49	30		50	29		40	39	
T4	13(3.6%)	10	3		9	4		6	7	
Inflammation				0.205			0.675			0.06
No	114(50.0%)	44	70		47	67		59	55	
Mild	97(42.5%)	48	49		46	51		53	44	
Severe	17(7.5%)	7	10		6	11		14	3	
Vascular invasion				0.051			0.034			0.615
No	200(65.8%)	88	112		84	116		108	92	
Yes	104(34.2%)	58	46		57	47		53	51	

Supplemental Table s3

Clinical features of 82 HCC patients based on UPB1, SOCS2 and RTN3 expression. The IHC score less than 1 was defined as the low expression group. The count data were analyzed with Pearson Chi-Square or Fisher's Exact Test. The quantitative data were analyzed with t-test or Mann-Whitney U test and the ordinal data were analyzed with nonparametric statistics as appropriate. Significance level: p<0.05.

Characteristics	Patients	UPB1 expression		p value	SOCS2 expression		p value	RTN3 expression		p value
		Low	High		Low	High		Low	High	
Gender				0.132			0.713			0.713
Male	74(90.2%)	43	31		37	37		37	37	
Female	8(9.8%)	2	6		5	3		5	3	
Age				0.255			0.048			0.488
Mean	56.37	55.11	57.89		58.69	53.93		57.19	55.5	
SD	10.96	10.12	11.86		11.82	9.52		11.61	10.3	
Tumor volume				0.819			0.167			0.83
Mean	100.59	75.64	127.56		118.88	79.75		114.91	85.1	
SD	175.94	111.3	222.7		200.37	143.16		211.08	128.86	
Tumor number				0.363			0.667			0.602
1	70(85.4%)	37	33		35	35		35	35	
2	5(6.1%)	3	2		4	1		3	2	
≥3	7(8.5%)	5	2		3	4		4	3	
Differentiation				0.727			0.119			0.747
Well-differentiated	5(6.1%)	4	1		1	4		3	2	
Moderate-differentiated	50(61.0%)	25	25		26	24		23	27	
Poor-differentiated	22(26.8%)	14	8		14	8		12	10	
Tumor capsule invaded				0.019			0.579			0.579
No	41(50.0%)	13	27		19	21		19	21	
Yes	41(50.0%)	24	17		22	19		22	19	
Micro-vessel invaded				0.892			0.699			0.925
No	57(69.5%)	31	26		30	27		29	28	
Yes	25(30.5%)	14	11		12	13		13	12	
T stage				0.543			0.882			0.565
T1	36(43.9%)	19	17		17	19		17	19	
T2	27(32.9%)	14	13		16	11		14	13	
T3	16(19.5%)	10	6		9	7		11	5	
T4	3(3.7%)	2	1		0	3		0	3	
Pathologic stage				0.698			0.736			0.445
Stage I	36(43.9%)	19	17		17	19		17	19	
Stage II	26(31.7%)	14	12		15	11		13	13	
Stage III	17(20.7%)	22	6		9	8		11	6	
Stage IV	3(3.7%)	1	2		1	2		1	2	
